# Antidiabetic effect of sciadonic acid on type 2 diabetic mice through activating the PI3K-AKT signaling pathway and altering intestinal flora

**DOI:** 10.3389/fnut.2022.1053348

**Published:** 2022-12-23

**Authors:** Lin Chen, Qihong Jiang, Hongling Lu, Chenkai Jiang, Wenjun Hu, Shaofang Yu, Xingwei Xiang, Chin Ping Tan, Yongcai Feng, Jianfang Zhang, Mingqian Li, Guoxin Shen

**Affiliations:** ^1^Institute of Sericultural and Tea, Zhejiang Academy of Agricultural Sciences, Hangzhou, Zhejiang, China; ^2^College of Food Science and Technology, Zhejiang University of Technology, Hangzhou, Zhejiang, China; ^3^Department of Food Technology, Faculty of Food Science and Technology, University Putra Malaysia, Serdang, Malaysia; ^4^Xujing (Hangzhou) Biotechnology Research Institute Co., Ltd., Hangzhou, Zhejiang, China; ^5^Cancer Institute of Integrated Traditional Chinese and Western Medicine, Zhejiang Academy of Traditional Chinese Medicine, Tongde Hospital of Zhejiang Province, Hangzhou, Zhejiang, China

**Keywords:** sciadonic acid, type 2 diabetes mellitus, insulin signaling pathway, gut microbiota, short-chain fatty acids

## Abstract

Type 2 diabetes mellitus (T2DM) is a metabolic disease characterized by hyperglycemia. The aim of this work was to investigate the effect of sciadonic acid (SA) on disorders of glucolipid metabolism and intestinal flora imbalance and to further investigate its potential molecular mechanism of anti-diabetes. The experimental data indicated that SA could alleviate hyperlipidemia, insulin resistance, oxidative stress, the inflammatory response, repair liver function damage, and promote glycogen synthesis caused by T2DM. SA could also activate the PI3K/AKT/GLUT-2 signaling pathway, promote glucose metabolism gene expression, and maintain glucose homeostasis. Furthermore, 16S rRNA analysis revealed that SA could reduce the Firmicutes/Bacteroidota (F/B) ratio; promote *norank_f__Muribaculaceae, Allobaculum, Akkermansia*, and *Eubacterium_siraeum_group* proliferation; increase the levels of major short-chain fatty acids (SCFAs), such as acetic acid, propionic acid, and butyric acid; and maintain the homeostasis of the intestinal flora. In conclusion, these results suggested that SA could reshape the structural composition of intestinal microbes, activate the PI3K/AKT/GLUT2 pathway, improve insulin resistance, and decrease blood glucose levels.

## 1 Introduction

Type 2 diabetes mellitus (T2DM) is a disorder of glucose metabolism characterized by increased insulin resistance leading to hyperglycemia (1). T2DM accounts for over 90% of people with diabetes and has become a major global public health problem (2). The global prevalence of diabetes among people aged 20–79 years is estimated to be 10.5% in 2021 and is expected to rise to 12.2% by 2045. The greatest increase in diabetes prevalence from 2021 to 2045 is predicted to occur in middle-income countries (21.1%), followed by high-income and low-income countries (12.2 and 11.9% respectively). By 2045, the number of adults with diabetes in middle-income countries will exceed 200 million (3). In particular, T2DM patients often suffer from complications due to hyperglycemia, including cardiovascular disease (4), kidney failure, and retinopathy (5). A variety of hypoglycemic drugs are currently used for the treatment of T2DM, including metformin (Met), thiazolidinediones, and sulfonylureas. However, the long-term use of these drugs can lead to certain side effects, such as gastrointestinal dysfunction (6), increased mortality, and cardiovascular risk (7). Therefore, researchers are increasingly committed to developing hypoglycemic substances with good therapeutic effects and no side effects.

Previous studies have found that vegetable oils rich in medium-chain fatty acids (MCFAs), which are fatty acids with carboxylic acid chains in the range of 6–12 carbons, may improve factors associated with T2DM by lowering total cholesterol (TC) and low-density lipoprotein (LDL) levels, reducing aspartate aminotransferase (AST) activity, and lowering the body weight (BW) and body mass index (BMI) (8, 9). *Torreya grandis* (*T. grandis*) is a large, evergreen, and ornamental coniferous tree belonging to the Cephalotaxaceae family and Torreya genus and mainly distributed in the hilly areas of subtropical China, especially in the Kuaiji Mountains of Zhejiang Province (10). *T. grandis* is known for its edible seeds with high nutritional and medicinal value. The seeds are rich in oils, fatty acids, proteins, vitamins, and mineral elements (10), which have biological activities, such as anti-oxidant (11), anti-tumor, and anti-inflammatory effects (12). The seed kernels of *T. grandis* contain approximately 42.6–61.5% oil. Unsaturated fatty acids were reported to account for 76.1–94.3% of the total fatty acids and to consist mainly of linoleic and oleic acids (13). Sciadonic acid (SA) is a polyunsaturated acid (5c, 11c, 14c-eicosatrienoic acid, SA) with anti-oxidant, anti-inflammatory, and improved lipid metabolic activities (14, 15). In the natural environment, SA is specifically abundant in conifers, such as edible pine seeds and *T. grandis* seeds. Compared with traditional oil, intragastric administration of *T. grandis* seeds oil containing SA could significantly improve obesity (14, 15). Nevertheless, the effect of SA on the alteration of gut microbiota and the potential mechanism through which it improves T2DM remain unclear. Therefore, in-depth studies on the hypoglycemic therapeutic effects of SA are important for refined production of *T. grandis* seeds and development of dietary supplements.

In the present study, we investigated the effects of SA on the PI3K/Akt signaling pathway at the gene and protein expression levels in a high-fat diet (HFD) and streptozotocin (STZ) induced T2DM mouse model. Furthermore, to investigate the possible mechanism of SA’s anti-diabetic activity, we also analyzed the effect of SA on the intestinal microbial structure and composition in T2DM mice. These results will help elucidate the hypoglycemic mechanism of SA, develop SA-related functional foods, and promote the high-value utilization of SA in the pharmaceutical industry.

## 2 Results

### 2.1 SA attenuated BW gain and insulin resistance in T2DM mice

**FIGURE 1:**
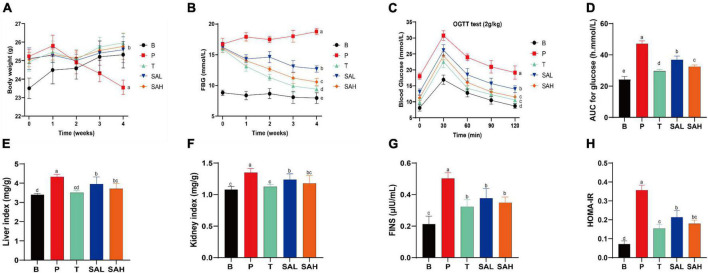
Sciadonic acid (SA) improved body parameters in type 2 diabetes mellitus (T2DM) mice. **(A)** BW, **(B)** FBG, **(C)** OGTT, **(D)** AUC of OGTT, **(E)** liver index, **(F)** kidney index, **(G)** FINS, and **(H)** HOMA-IR. Data were presented as the mean ± SD (*n* = 6). Different letters represent a significant difference among multiple groups (*P* < 0.05). B, control group; P, diabetic model group; T, (HFD + 200 mg kg^– 1^ Met); SAL, (HFD + 70 mg kg^– 1^ SA); SAH, (HFD + 280 mg kg^– 1^ SA).

Type 2 diabetes mellitus caused weight loss and disrupted organismal function. In the early stages of diabetes, the BW of mice increased significantly in all groups. However, the weight gain was 7.74% in group B mice, and the weight loss was 6.67% in group P mice compared to the beginning of the study. Notably, SA could inhibit the weight loss trend in T2DM mice in a dose-dependent manner and was not significantly different from that in normal mice (Figure 1A). In addition, the persistent hyperglycemia resulted in significantly increased liver and kidney indices in T2DM mice compared with the mice in group B, while the indices were significantly decreased in the T, SAL, and SAH groups; notably, SAH was more effective (Figures 1E, F). The experimental results revealed that HFD caused obesity along with abnormal blood glucose levels. The initial FBG levels in the HFD/STZ-induced T2DM mouse group were significantly higher than those in the B group. Compared with the P group, the FBG of mice in the T, SAL, and SAH groups decreased significantly after 4 weeks of Met and SA treatment (*P* < 0.05) by 41.01, 21.61, and 31.14%, respectively (Figure 1B).

To further analyze the tolerance of mice to blood glucose, we used the OGTT. In Figures 1C, D, the OGTT of blood glucose over 120 min showed a flatter overall blood glucose curve in group B. While the curve was steeper and the AUC increased significantly in group P, indicating that glucose tolerance was impaired in T2DM mice. After the Met and SA interventions, blood glucose levels exhibited a strong and significant decrease compared to the P group, indicating that SA may improve glucose tolerance in diabetic mice. Furthermore, insulin levels were significantly higher in T2DM mice compared to group B (*P* < 0.05), demonstrating the development of insulin resistance (Figure 1G). Met and SA administration induced FINS reductions of 55.25, 33.02, and 43.94% in the T, SAL, and SAH groups, respectively. HOMA-IR values were used to reveal the ability of insulin to transform glucose in mice. The greater the HOMA-IR, the lower the insulin sensitivity. And the greater the insulin resistance, and the lower the hypoglycemic efficiency. In the present work, T2DM mice became severely insulin resistant, with HOMA-IR values significantly higher than those in group B (*P* < 0.05). HOMA-IR was significantly lower in the SAL and SAH groups than in the P group and decreased by 40.02 and 49.56%, respectively (Figure 1H). These results suggested that SA could improve HFD/STZ-induced hyperglycemia and might play an important role in the hypoglycemic effect.

### 2.2 SA improved lipid metabolic disorders, oxidative stress, and inflammation in T2DM mice

**FIGURE 2:**
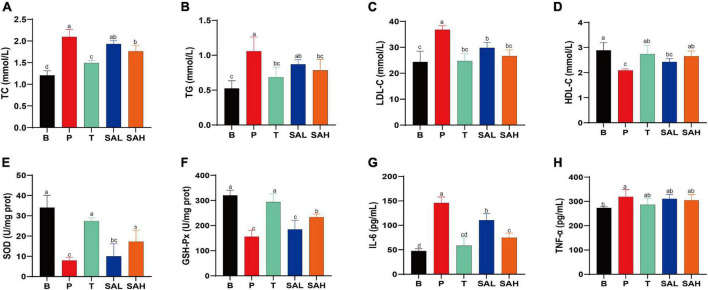
Sciadonic acid (SA) regulated the serum metabolic parameters in type 2 diabetes mellitus (T2DM) mice. **(A)** TC, **(B)** TG, **(C)** LDL-C, **(D)** HDL-C, **(E)** SOD, **(F)** GSH-Px, **(G)** IL-6, and **(H)** TNF-a. Data are presented as the mean ± SD of three experiments. Different letters represent a significant difference among multiple groups (*P* < 0.05). B, control group; P, diabetic model group; T, (HFD + 200 mg kg^– 1^ Met); SAL, (HFD + 70 mg kg^– 1^ SA); SAH, (HFD + 280 mg kg^– 1^ SA).

To investigate the effect of SA on lipid metabolism, TC, TG, LDL-C, and HDL-C levels in mouse serum were measured. The results showed that TG, TC, and LDL-C levels were significantly higher, whereas HDL-C levels were lower in the P group than in the B group. Met and SA treatment significantly improved these indices compared to the P group. Thus, these findings suggested that the intervention with SA improved the dyslipidemia in T2DM mice (Figures 2A–D). Previous studies demonstrated that oxidative stress plays a causal role in T2DM (16). The activity levels of SOD and GSH-Px in the serum of mice in group P were lower than those in group B (*P* < 0.05) (Figures 2E, F). In contrast, the levels of SOD and GSH-Px activities in the liver of mice in the SA group were significantly higher (*P* < 0.05) and showed a dose-dependent pattern. This indicated that SA could improve the antioxidant capacity of T2DM mice. Besides, our research data demonstrated that SA could reduce the serum levels of IL-6 and TNF-α in T2DM mice (Figures 2G, H), with a more significant effect in reducing IL-6 levels (*P* < 0.05).

### 2.3 SA reduces hepatic steatosis in STZ-induced T2DM mice

**FIGURE 3:**
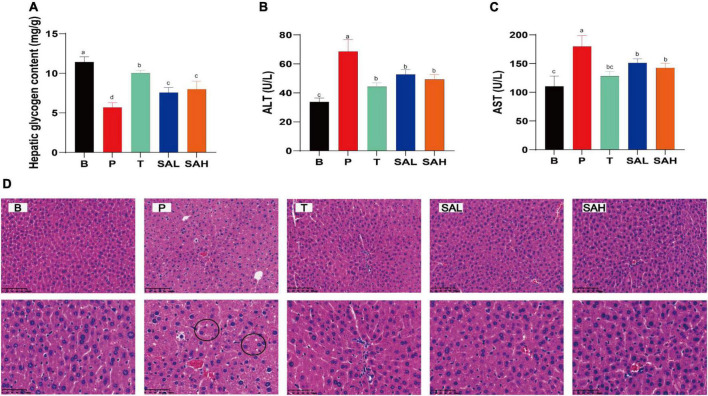
Sciadonic acid (SA) ameliorates hepatic impairment in type 2 diabetes mellitus (T2DM) mice. **(A)** Hepatic glycogen content, **(B)** ALT, **(C)** AST, and **(D)** H&E stain. Data are presented as the mean ± SD of three experiments. Different letters represent a significant difference among multiple groups (*P* < 0.05). B, control group; P, diabetic model group; T, (HFD + 200 mg kg^– 1^ Met); SAL, (HFD + 70 mg kg^– 1^ SA); SAH, (HFD + 280 mg kg^– 1^ SA).

Studies have shown that T2DM is often associated with metabolic-associated fatty liver disease (MAFLD) and cardiovascular disease (17). Hepatic glycogen levels were significantly lower (*P* < 0.05), while AST and ALT activities were significantly increased (*P* < 0.05) in mice in the P group compared with those in the B group. This indicated that liver function was impaired in T2DM mice, weakening the glucose conversion capacity. However, AST and ALT activities were significantly reduced in the serum of SAL and SAH mice (*P* < 0.05), and liver glycogen levels were significantly increased after 4 weeks of SA administration (Figures 3A–C). To further evaluate the effect of SA on liver tissue integrity in T2DM mice, H&E staining was used. H&E staining of the liver tissue is shown in Figure 3D. The hepatocytes in group B mice were structurally normal, neatly arranged, and with obvious nuclei and nucleoli, whereas the hepatocytes of mice in group P were disordered, increased in size, and contained fat vesicles. Moreover, the mice in group P had erythrocytes in the lumen and a small number of inflammatory cells infiltrated around the veins. Compared with T2DM mice, Met and SA improved the above pathology, reduced the number of vacuoles in hepatocytes, and the hepatocytes more closely resembled those of healthy mice. In summary, SA may restore liver tissue injury and improve liver function to promote glycogen synthesis.

### 2.4 SA promoted carbohydrate metabolism gene expression

**FIGURE 4:**
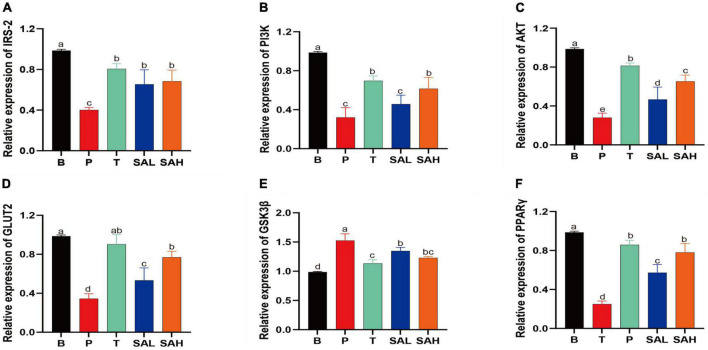
Sciadonic acid (SA) promotes gluconeogenic gene expression. Data are presented as the mean ± SD of three experiments. **(A)** IRS-2, **(B)** PI3K, **(C)** AKT, **(D)** GLUT-2, **(E)** GSK3ß, and **(F)** PPARγ. Data are presented as the mean ± SD of three experiments. Different letters represent a significant difference among multiple groups (*P* < 0.05). B, control group; P, diabetic model group; T, (HFD + 200 mg kg^– 1^ Met); SAL, (HFD + 70 mg kg^– 1^ SA); SAH, (HFD + 280 mg kg^– 1^ SA).

In the present study, the mRNA expression levels of IRS-2, PI3K, AKT, GLUT-2, GSK3β, and PPAR-γ were determined using real-time quantitative PCR (Figure 4). The expression of IRS-2, PI3K, AKT, GLUT-2, and PPAR-γ was significantly decreased (*P* < 0.05), whereas the expression of GSK3β was significantly increased in the P group compared to the B group. After 4 weeks of SA treatment, the levels of IRS-2, PI3K, AKT, GLUT-2, and PPAR-γ were significantly increased, while the relative expression of GSK3β was decreased. These data suggested that SA could exert hypoglycemic effects by activating glucose metabolism signaling genes, such as PI3K/AKT/GLUT-2.

### 2.5 SA activated the expression of PI3K/AKT pathway-related proteins

**FIGURE 5:**
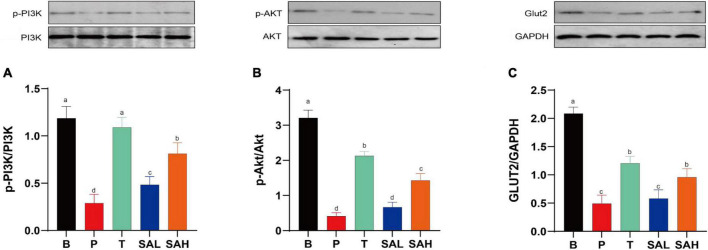
Sciadonic acid (SA) improved type 2 diabetes mellitus (T2DM) by activating the hepatic PI3K/AKT/GLUT-2 signaling pathway. **(A)** p-PI3K/PI3K, **(B)** p-AKT/AKT, and **(C)** GLUT-2/GAPDH. Data are presented as the mean ± SD of three experiments. Different letters represent a significant difference among multiple groups (*P* < 0.05). B, control group; P, diabetic model group; T, (HFD + 200 mg kg^– 1^ Met); SAL, (HFD + 70 mg kg^– 1^ SA); SAH, (HFD + 280 mg kg^– 1^ SA).

Phosphorylation is a critical pathway for the activation of insulin-signaling proteins. The expression of p-PI3K/PI3K, p-AKT/AKT, and GLUT-2 proteins was significantly decreased (*P* < 0.05) in the P group compared to the B group as determined with western blotting (Figure 5). p-PI3K/PI3K expression was significantly increased in the SAL group compared with the P group (*P* < 0.05). In addition to p-PI3K, the expression of p-AKT/AKT and GLUT-2 was also increased in the SAL group, although not significantly. Moreover, a high dose of SA was more effective in activating the expression of p-PI3K/PI3K, p-AKT/AKT, and GLUT-2 in T2DM mice (*P* < 0.05).

### 2.6 SA increased intestinal flora diversity in T2DM mice

**FIGURE 6:**
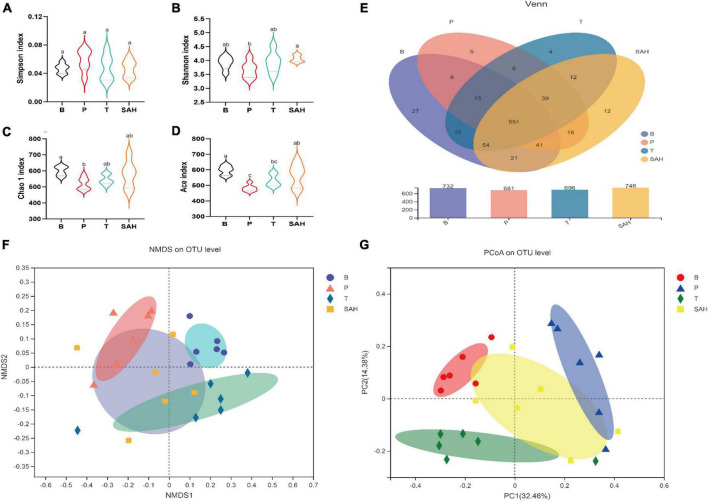
Sciadonic acid (SA) relieved gut microbiota disturbances caused by type 2 diabetes mellitus (T2DM). **(A)** Simpson index, **(B)** Shannon imdex, **(C)** Chao 1 index, **(D)** ace index, **(E)** venn diagram, **(F)** NMDS, and **(G)** PCoA analysis diagram. Data were presented as the mean ± SD (*n* = 6). Different letters represent a significant difference among multiple groups (*P* < 0.05). B, control group; P, diabetic model group; T, (HFD + 200 mg kg^– 1^ Met); SAH, (HFD + 280 mg kg^– 1^ SA).

Fatty acids have been reported to improve insulin resistance and to play an important role in glucose/insulin metabolism (18). To further investigate whether the anti-diabetic effect of SA was related to the intestinal microbiota, we analyzed the microbiota in the cecum of the mice in each group. Valid sequences were clustered into operational taxonomic units (OTUs) based on 97% similarity. A total of 907,992 valid sequences from 24 samples (*n* = 6) with 820 different OTUs were obtained. The results of rank-abundance, pan/core, Shannon, and Simpson rarefaction curves (Supplementary Figure 1) indicated that the number of sequencing samples was sufficient. Compared with the P group, the Shannon, Chao1, and Ace indices increased significantly, and the Simpson index decreased significantly in the SAH group, indicating that SA could increase the richness and diversity of the microbial community (Figures 6A–D). A Venn diagram (Figure 6E) revealed 732, 681, 696, and 746 OTUs for the B, P, T, and SAH groups, respectively. The number of reciprocal OTUs for all samples was 551, with a total of 615 for the B and P groups and 667 for the B and SAH groups. Notably, the number of OTUs specific to the B and P groups was 21 and that for the B and SAH groups was eight. β-Diversity analysis, including non-metric multidimensional scaling analysis (NMDS) and principal coordinate analysis (PCoA), indicated that the structural composition of the microbial community was completely separated between the P and B groups and suggested that the structure of the HFD/STZ-induced intestinal flora in T2DM mice had been disrupted. However, SA could restore the intestinal flora closer to the normal group (Figures 6F, G).

### 2.7 SA improved the intestinal flora composition in T2DM mice

**FIGURE 7:**
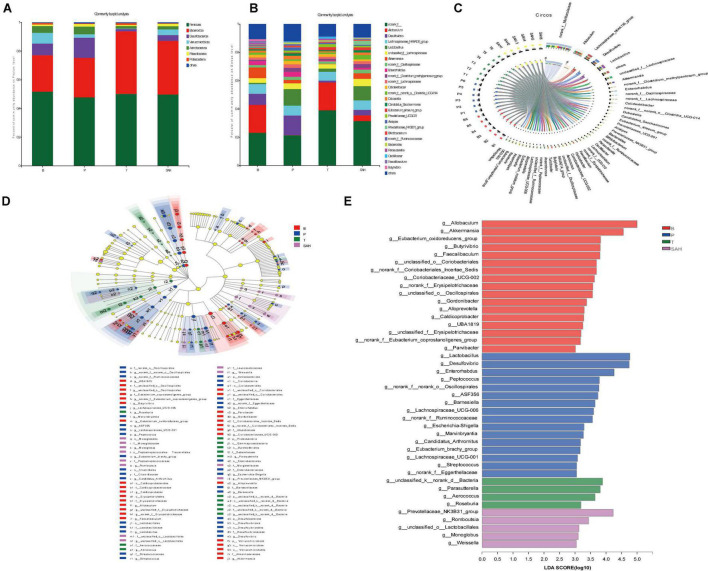
Sciadonic acid (SA) regulated intestinal flora composition in type 2 diabetes mellitus (T2DM) mice. **(A)** Percent community abundance diagram on phylum level, **(B)** percent community abundance diagram on genus level, **(C)** in Circo’s graphs, the left semicircle represents the genera composition, and the right semicircle indicated the distribution of each genus in the experimental groups, **(D)** lEfSe multi-level classification tree diagram, and **(E)** LDA discriminant histogram. Data were presented as the mean ± SD (*n* = 6). B, control group; P, diabetic model group; T, (HFD + 200 mg kg^– 1^ Met); SAH, (HFD + 280 mg kg^– 1^ SA).

To further assess the effect of SA on the intestinal flora, we analyzed the intestinal microbiota composition of each group at the phylum and genus levels. Bacteroidetes and Firmicutes were the main dominant phyla, accounting for more than 75% of the total microbiota. Compared to group B, the relative abundance of Firmicutes and Verrucomicrobiota in group P decreased by 7.67 and 68.20%, respectively, while the relative abundance of Bacteroidota and Desulfobacterota increased by 8.09 and 74.33%, respectively. The relative abundance of Firmicutes/Bacteroidota was 1.73. Compared to the P group, the Met intervention significantly increased the relative abundance of Firmicutes and Bacteroidota but decreased the relative abundance of Desulfobacterota and Verrucomicrobiota. After 4 weeks of SA gavage, the relative abundance of Firmicutes, Bacteroidota (F/B = 1.32), and Verrucomicrobiota increased by 4.11, 36.17, and 65.56%, respectively, while the relative abundance of Desulfobacterota and Actinobacteriota decreased by 71.59 and 59.03%, respectively (Figure 7A).

To further investigate the differences in intestinal microbiota between the B, P, T, and SAH groups, the abundance of 26 groups at the genus level was analyzed (Figure 7B). *Norank_f__Muribaculaceae, Allobaculum*, the *Lachnospiraceae_NK4A136_group, Desulfovibrio*, and *Lact obacillus* were the most dominant genera in all groups (Figure 7C). The linear discriminant analysis effect size (LEfSe) (Figure 7D) and linear discriminant analysis (LDA) (Figure 7E) were used to analyze the differentially abundant taxa in the different groups. The microbial communities in groups B and SAH belonged mainly to Firmicutes and Bacteroidetes, but with different genus types. In particular, the B group mainly showed a significant selective enhancement of *Allobaculum, Akkermansia*, and the *Eubacterium_oxidoreducens_group* for the genus, while *Lactobacillus, Desulfovibrio*, and *Enterorhabdus* were the most represented bacteria in the T2DM group. The intervention markers of the SAH group were the *Prevotellaceae_NK3B31_group* and *Romboutsia* genera. Overall, these results suggested that SA altered the gut microbiome of T2DM mice.

**FIGURE 8:**
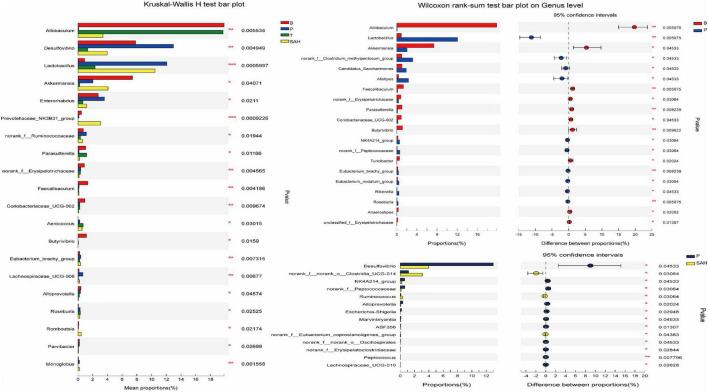
Comparisons between different groups at the genus level. *y*-axis indicates species name at a given taxonomic level, *x*-axis indicates mean relative abundance in different groups of species, different colors indicate different groups; rightmost *P*, *0.01 & *P* ≤ 0.05, **0.001 & *P* ≤ 0.01, ***P ≤ 0.001. Data were presented as the mean ± SD (*n* = 6). B, control group; P, diabetic model group; T, (HFD + 200 mg kg^– 1^ Met); SAH, (HFD + 280 mg kg^– 1^ SA).

The intestinal flora dysbiosis in T2DM mice was represented by the fact that *norank_f__Muribaculaceae, Allobaculum, Akkermansia, norank_f__norank_o__Clostridia_UCG-014*, the *Eubacterium_siraeum_group*, and *Prevotellaceae_NK3B31_ group* were lower in abundance, while *Desulfovibrio, Enterorhabdus*, the *norank_f__Clostridium_methylpentosum_ group, Dubosiella, Alistipes*, and *Bacteroides* were higher in abundance. Based on the significant alterations in intestinal flora caused by T2DM, we found that all nine genera could be reversed by Met and SA treatment, including significantly increased levels of *norank_f__Muribaculaceae, Allobaculum*, and the *Eubacterium_siraeum_group*, as well as significantly decreased levels of *Desulfovibrio, Enterorhabdus*, the *norank_f__Clostridium_methylpentosum_group, Dubosiella, Alistipes*, and *Bacteroides*. Besides, the relative abundance of *Akkermansia, norank_f__norank_o__Clostridia_UCG-014*, and the *Prevotellaceae_NK3B31_group* increased significantly after SA treatment (Figure 8).

### 2.8 SA increased the cecum content concentration of SCFAs in T2DM mice

**FIGURE 9:**
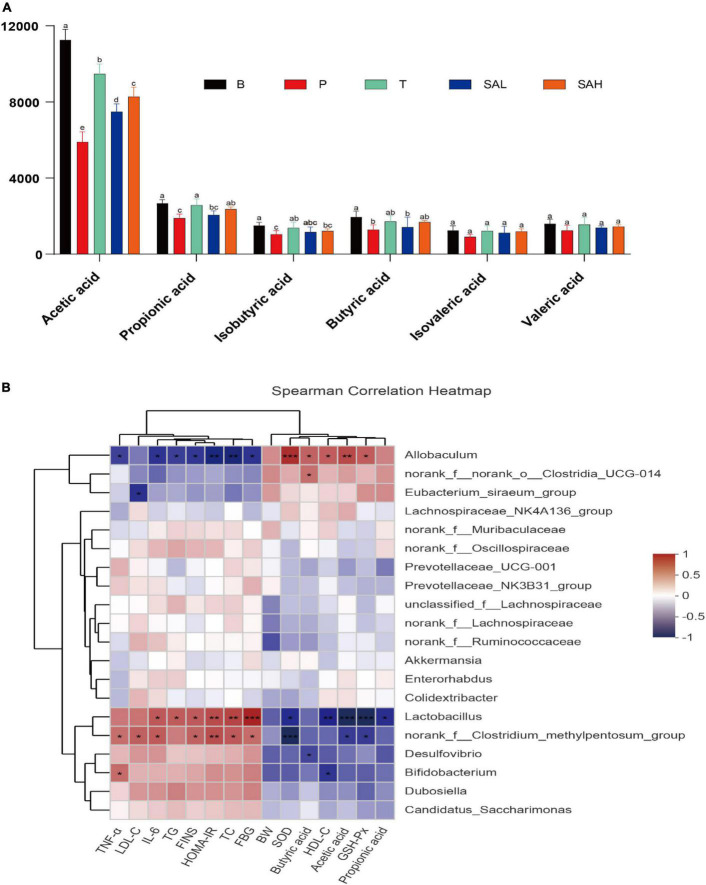
Correlation analysis between biochemical parameters and intestinal microbiota at the genus level. **(A)** SCFAs, **(B)** Spearman’s correlation analyses were used to analyze the relative abundance between the specific genus in each group. Data were presented as the mean ± SD (*n* = 6). Groups with different letters are significantly different (*P* < 0.05). B, control group; P, diabetic model group; T, (HFD + 200 mg kg^– 1^ Met); SAL, (HFD + 70 mg kg^– 1^ TGP); SAH, (HFD + 280 mg kg^– 1^ TGP).

The concentrations of acetic acid, propionic acid, isobutyric acid, butyric acid, isovaleric acid, and valeric acid are shown in Figure 9A. The levels of primary SCFAs, such as acetic acid, propionic acid, and butyric acid, were significantly lower in group P than in group B (*P* < 0.05). The levels of acetic acid, propionic acid, butyric acid, and isobutyric acid were significantly higher in the T group than in the P group (*P* < 0.05). Furthermore, the concentrations of acetic acid and propionic acid were significantly higher in the SAL and SAH groups than in the P group after an SA gavage (*P* < 0.05), and the concentrations of isobutyric acid, n-butyric acid, isovaleric acid, and n-valeric acid were slightly but not significantly increased.

### 2.9 Correlation between biochemical parameters and intestinal microbiota

Correlations between gut microbes and biochemical indicators in the samples were demonstrated by Spearman correlation analysis. As shown in Figure 9B, TNF-α, IL-6, LDL-C, TG, TC, FBG, FINS, and HOMA-IR biochemical indicators were positively correlated with *Lactobacillus*, the *norank_f__Clostridium_methylpentosum_group, Desulfovibrio, Bifidobacterium, Dubosiella*, and *Candidatus_Saccharimonas*, while they were negatively correlated with *Allobaculum, norank_f__norank_o__Clostridia_UCG-014*, and the *Eubacterium_siraeum_ group*. In addition, *Akkermansia* and *Colidextribacter* were negatively correlated with FINS and HOMA-IR. However, BW, HDL-C, SOD, GSH-Px, butyric acid, acetic acid, and propionic acid were positively correlated with *Allobaculum, norank_f__norank_o__Clostridia_UCG-014*, and the *Eubacterium_siraeum_group*, while they were negatively correlated with *Lactobacillus*, the *norank_f__ Clostridium_methylpentosum_group, Desulfovibrio*, and *Bifidobacterium*. These relationships suggest that biochemical indicators, metabolites, and gut microbiota can interact with each other.

## 3 Discussion

*T. grandis* oil is a highly valuable oil product rich in a variety of unsaturated fatty acids, including oleic acid, linoleic acid, and SA. Previous studies have shown that *T. grandis* oil has anti-tumor and anti-hyperlipidemic properties (15). However, little has been reported in the literature about the effect of SA on the intestinal flora of patients with T2DM and the possible mechanisms of hypoglycemia. Moreover, further work is needed to obtain information about the interaction between SA and T2DM hosts in order to develop functional foods related to SA. In current study, we have demonstrated that SA improves glucose metabolism disorder *via* the regulation of PI3K/Akt pathway and intestinal microbiota -mediated glycogen synthesis and glucose metabolism in HFD/STZ-induced T2DM mice.

The results of the current investigation indicated that HFD/STZ-induced T2DM mice lost weight and had markedly elevated liver index, kidney index, and FBG levels, which may be caused by disturbances in lipid and glucose metabolism. The increased lipolysis, elevated levels of free fatty acids, and accumulation of intermediate lipid metabolites increased the glucose output, decreased peripheral glucose utilization, and impaired β-cell function (19). Further, mice in the P group lost 6.67% of their BW compared to that at the beginning of the study, and the trend of weight loss might intensify with the development of diabetes. However, after 4 weeks of gavage, we found that SA effectively inhibited weight loss and FBG elevation (Figures 1A, B) in a dose-dependent manner. This may be due to the fact that SA improved insulin sensitivity and reduced blood glucose levels in T2DM mice. Subsequent OGTT results also supported this notion, with high doses of SA significantly improving glucose tolerance in T2DM mice (Figures 1C, D), lowering blood insulin levels (Figure 1G), ameliorating insulin resistance (Figure 1H), and promoting glucose metabolism. Impaired glucose tolerance is an intermediate state in the transition from normal glucose tolerance to T2DM (20). In our experiments, we observed that the FBG of individual SA-treated T2DM mice was close to that of normal mice and that SA treatment could enhance glucose tolerance.

Hyperlipidemia is one of the characteristics of T2DM, mainly manifesting with significantly increased levels of TC, TG, and LDL-C but decreased levels of HDL-C. Increased levels of TC and TG tend to increase the risk of cardiovascular disease in patients with T2DM (21). SA could significantly reduce TC, TG, and LDL-C levels in the serum of T2DM mice, promote HDL-C production, and improve hyperlipidemia (Figures 2A–D). In addition, recent studies have demonstrated that oxidative stress disrupts cellular structures and manipulates signaling pathways, which are believed to be a key mechanism of insulin resistance (22). In the present study, SA significantly increased the of SOD levels and GSH-Px in the serum of T2DM mice and enhanced the antioxidant capacity (Figures 2E, F). Meanwhile, SA reduced IL-6 and TNF-α levels and attenuated the inflammatory response. It has been reported that IL-6 and TNF-α inhibit insulin receptor signaling in the liver of T2DM mice, thus reducing insulin sensitivity and impeding glucose uptake and utilization (23).

The results of H&E staining analysis showed that the liver tissues of T2DM mice were damaged, and the markers of liver function impairment (ALT and AST) were significantly increased, whereas the liver glycogen content was significantly decreased. This could be due to the oxidative stress and inflammatory response that impair liver function in T2DM mice, resulting in reduced hepatic glycogen synthesis and inability to properly take in and utilize glucose (Figure 3). Furthermore, the PI3K/Akt pathway is strongly associated with insulin resistance signaling, and changes in its expression have an important role in regulating glucose metabolism (24). RT-PCR and western blot analysis revealed that SA supplementation promoted the expression of IRS-2, PI3K, AKT, GLUT2, and PPARγ glucose metabolism-related genes in liver tissue, and inhibited GSK3β gene expression (Figure 4). In addition, SA supplementation activated the PI3K/Akt signaling pathway and significantly up-regulated the protein expression of p-PI3K/PI3K, p-AKT/AKT, and GLUT2 in the liver of hyperglycemic mice, thereby ameliorating hepatic metabolic disorders (Figure 5). GLUT2 expression plays a key role in glucose sensing and homeostasis, and its inactivation in the liver could impair glucose-stimulated insulin secretion (25). GSK3β was reported to be significantly increased in patients with T2DM and was positively correlated with insulin resistance (26). In addition, PPARγ is a ligand-activated nuclear receptor, and activation of PPARγ improves insulin sensitivity (27). In summary, SA could activate the PI3K/AKT/GLUT2 protein pathway, regulate glucose metabolism gene expression, enhance insulin sensitivity, and improve insulin resistance.

Numerous studies have demonstrated that the gut microbiota are associated with the development of T2DM and related complications, which can regulate glucose homeostasis to alleviate hyperglycemia (28). The results of gut microbiotic analysis indicated that SA significantly reduced the F/B ratio at the gate level, and the F/B ratio was reported to be negatively correlated with glucose levels and tolerance (29). Meanwhile, the relative abundance of Desulfobacterota and Actinobacteriota was significantly reduced. It was previously shown that the abundance of Desulfobacterota was positively correlated with BW and lipid concentration (30), which is consistent with the above results (Figure 1). Furthermore, the induction of T2DM mice using HFD/STZ caused a significant decrease in the abundance of *norank_f__Muribaculaceae, Allobaculum, Akkermansia, norank_f__norank_o__Clostridia_UCG-014*, the *Eubacterium_siraeum_ group*, and *Prevotellaceae _NK3B31_group*, while the abundance of *Desulfov ibrio, Lactobacillus, Enterorhabdus, norank_f__Clostridium _methylpentosum_group, Dubosiella, Alistipes*, and *Bacteroides* was significantly increased. At the genus level, supplementation with high doses of SA significantly reversed their abundance (Figure 7). *Norank_f_Muribaculaceae* are reportedly widespread in the intestinal flora and are functionally diverse in degrading carbohydrates (31). Recent studies have demonstrated that higher abundance of Muribaculaceae positively correlates with the activation of the PI3K/Akt signaling pathway (32). *Allobaculum* was identified as an active glucose assimilator, whose main metabolites are acetic acid and propionic acid (33) and which has a positive effect on the regulation of the intestinal flora ecosystem. *Akkermansia* maintains the integrity of the mucin layer and has anti-inflammatory and insulin resistance improving properties (28). However, SA significantly reduced the relative abundance of *Desulfovibrio, Enterorhabdus*, the *norank_f__Clostridium_methylpentosum_group, Dubosiella*, and *Alistipes*. Notably, *Desulfovibrio, Enterorhabdus*, and *Dubosiella* were positively correlated with intestinal inflammation and oxidative stress (34, 35), which is consistent with the experimental results showing a significant increase in IL-6 and TNF-α and a significant decrease in SOD and GSH-Px in T2DM mice (Figure 2).

Regulation of the intestinal flora and SCFA levels in T2DM mice had a positive effect on improving hyperglycemia (36). A correlation analysis indicated that the abundance of gut microflora was significantly correlated with metabolic parameters, including the lipid concentration, FBG, FINS, HOMA-IR, inflammatory markers, antioxidant enzymes, and SCFAs. Several studies have demonstrated that SCFAs activate the PI3K/AKT signaling pathway and alleviate T2DM (37–39). Therefore, we hypothesized that the modulatory effect of SA on glycolipid metabolism could be attributed to the reduced abundance of these harmful pathogenic bacteria and increased abundance of beneficial bacteria, promoting the production of SCFAs, and alleviate T2DM symptoms.

## 4 Conclusion

In our work, we found that administration of SA to HFD/STZ-induced T2DM mice significantly inhibited weight loss and the FBG level while increasing insulin sensitivity and improving insulin resistance. Furthermore, SA reduced TC, TG, and LDL-C levels and increased HDL-C levels, as well as reduced fat accumulation in liver tissues, enhanced glucose uptake and utilization by liver tissues, and promoted glycogen synthesis. Meanwhile, SA could activate the PI3K/AKT signaling pathway, promote glucose metabolism gene expression, and regulate blood glucose. Notably, the SA intervention altered the composition of the gut microbiota in T2DM mice and increased the levels of SCFAs, such as acetate, propionate, and butyrate. In conclusion, our experimental results showed that SA could produce hypoglycemic effects by regulating gut microbial structure and activating the PI3K/AKT signaling pathway. Our research supports the potential of SA as a therapeutic agent.

## 5 Materials and methods

### 5.1 Chemicals and reagents

*T. grandis* cv. *Merrillii* was purchased from Zhuji (Zhejiang, China), and SA was extracted according to a conventional method (40). STZ and Met were obtained from Aladdin Reagent Int. (Shanghai, China). HFD feed was purchased from Suzhou Shuangsi Experimental Animal Feed Technology Co. (Suzhou, China). The other chemical reagents were of analytical grade.

### 5.2 Animal experiments

Thirty male SPF C57BL/6 J mice (weighing 24 ± 2 g) aged 7 weeks were purchased from Hangzhou Ziyuan Experimental Animal Science and Technology Co., Ltd. (certificate number SCXK (zhe) 2019-0004, Hangzhou, China). All mice were housed in a non-pathogenic animal chamber maintained at 23–25°C, 50–60% relative humidity, and 12/12 h of dark/light illumination. After a week, the mice were randomly divided into two groups, a control group (B, *n* = 6) and an HFD group (*n* = 24). After 3 weeks of HFD feeding, the 24 mice were fasted overnight and intraperitoneally injected with 100 mg/kg BW of STZ (0.1 M cold citrate buffer, pH 4.5), whereas 0.1 M cold citrate buffer was administered to the control group. The blood glucose level of the mice was stable after a week. Subsequently, blood was taken from the mouse tail veins, and the level of fasting blood glucose (FBG) was measured. An FBG level > 11.1 mmol/L was considered to indicate T2DM (41), and the mice were used for further research.

The type 2 diabetic mice were randomly divided into four groups, with six animals in each group: a model group (P), a positive control group (T, 200 mg/kg BW Met), a low-dose group (SAL, 70 mg/kg BW SA), and a high-dose group (SAH, 280 mg/kg SA). The mice in the control and model groups were administered the same amount of distilled water every day for 4 weeks. BW, food intake, and FBG were measured weekly. All mice were fed a normal diet of regular chow. After 4 weeks of treatment, the mice were fasted overnight and were then killed under iso-flurane anesthesia. Blood samples were collected and centrifuged at 3,000 rpm for 10 min to obtain serum, which was stored at −80°C. Liver samples were collected and divided into two parts for hematoxylin & eosin (H&E) staining and western blot analysis. Cecal content was collected for the detection of short-chain fatty acids (SCFAs) and microorganisms. The formula for calculating the liver/kidney index is as follows:


$$\mathrm{Organ}\mathrm{index}=\frac{Organweight}{Bodyweight}$$

### 5.3 FBG and oral glucose tolerance test (OGTT)

During the experiment, blood was drawn from the tail vein of mice once a week after overnight fasting to detect FBG levels. After 3 weeks of gavage, all mice were fasted overnight and then given glucose (2 g/kg BW) dissolved in water (42). The blood glucose levels were measured using blood obtained from the tail vein at 0, 30, 60, 90, and 120 min with a blood glucometer (Sannuo, Beijing, China).

### 5.4 Measurement of serum insulin level and insulin resistance

According to the kit instructions, the fasting serum insulin levels (FINS) were measured by using an insulin detection kit (Nanjing Jiancheng Bioengineering Co. Ltd., Nanjing, China). The insulin resistance (HOMA-IR) was calculated as follows (43):


$$HOMA-IR=\frac{insulin\left(\mu IU/mL\right)\times glucose\left(\mu mol/mL\right)}{22.5}$$

### 5.5 Determination of serum lipids

The triglyceride (TG), total cholesterol (TC), high-density lipoprotein cholesterol (HDL-C), and LDL cholesterol (LDL-C) levels in the serum were determined using an ELISA kit (Nanjing Jiancheng Bioengineering Co. Ltd., Nanjing, China).

### 5.6 Evaluation of inflammation and oxidative stress parameters

The serum levels of superoxide dismutase (SOD), glutathione peroxidase (GSH-Px), tumor necrosis factor-α (TNF-α), and interleukin-6 (IL-6) were measured according to the kit instructions. SOD and GSH-Px kits were purchased from Jiancheng Bioengineering Institute (Nanjing, China). TNF-α and IL-6 kits were obtained from Wuhan Boster Biological Technology., Ltd. (Wuhan, China).

### 5.7 Determination of ALT, AST, and hepatic glycogen

The levels of alanine transaminase (ALT), AST, and hepatic glycogen (Gly) were measured following the instructions on the ELISA kits (Nanjing Jiancheng Bioengineering Co. Ltd., Nanjing, China).

### 5.8 H&E staining of liver tissues

Liver specimens from mice in each group were fixed in 4% formaldehyde, embedded in paraffin, and sectioned for H&E staining. The liver tissue changes were observed under a light microscope.

### 5.9 Real-time quantitative RT-PCR analysis

RT-PCR analysis was performed according to previously published research methods (44). Briefly, total RNA was extracted from mouse liver tissues using Trizol reagent (Tiangen Biotech Co., Ltd., Beijing, China) and quantified by optical density measurements at 260 and 280 nm using a spectrophotometer (Tiangen Biotech Co., Ltd., Beijing, China). cDNA was synthesized from RNA samples with a cDNA synthesis kit (Tiangen Biotech Co., Ltd., Beijing, China). Each sample was analyzed using qRT-PCR with the SYBR Green PCR Master Mix (Tiangen Biotech Co., Ltd., Beijing, China) and quantified utilizing the StepOnePlus RT-PCR detection system (Zhejiang Scientific Instruments and Materials I/E Co., Ltd.). β-Actin was used as a control housekeeping gene for calculation of the relative gene expression levels. The primer sequences used for PCR were synthesized by Shanghai Sangon Biotech Co., Ltd., China (Table 1).

**TABLE 1:** Sequences of oligonucleotide primers used in qRT-PCR analysis.

Primer name	Sequence (5′–3′)	
	Forward	Reverse
SOD	TGGAGGCCACATCAATCATA	AGCGGTCAACTTCTCCTTGA
GSH-Px	TCGTGGCTTCCCTTGCAAC	CCATTCACGTCACACTTCTG
TNF-α	CACCTCAGACAAAATGCTCTTCAC	CTCACACATCTCCTTTCTCATTGC
IL-6	CAGGTCTATTTTGGGATCATTGCC	TCCCTGATTTCTAAGTGTTGCTGT
IRS-2	CCACCATCGTGAAAGAGTGA	TTGCCTTGTTGGTGCCTCAT
PI3K	ACAGGCACAACGACAACATC	TAAGCCCTAACGCAGACATC
AKT	TTTGGGAAGGTGATCCTGGTG	GGTCGTGGGTCTGGAATGAGT
GSK-3β	TAGTCCGATTGCGGTATTT	GGAATGGATATAGGCTAGACT
GLUT-2	ATGAACCCAAAACCAACCCCT	GGCCTGAAATTAGCCCTTCCA
PPAR-γ	TTACCACGGTTGATTTCTC	GACGCAGGCTCTACTTTGAT
β-actin	AGTGTGACGTTGACATCCGT	GCAGCTCAGTAACAGTCCGC

### 5.10 Western blotting

Mouse liver tissues were crushed in liquid nitrogen, and total protein was extracted using a total protein extraction kit (containing a protease inhibitor cocktail), followed by total protein quantification utilizing a BCA quantitation kit. All proteins were separated using 8–12% SDS-PAGE gels and blotted on PVDF membranes. Next, the membranes were placed in T-TBS (with 5% BSA) and blocked for 1 h at room temperature. Then, the membranes were rinsed in T-TBS and incubated with primary and secondary antibodies. The relative expression of target proteins was calculated using GAPDH as an internal reference. Image J software was employed to analyze the densitometric values of the bands and to calculate the relative expression of the proteins of interest. The primary antibodies used in this study are shown in Table 2.

**TABLE 2:** Primary antibody information in western blotting experiments.

Primary antibody name	Brand and catalog no	Dilution	molecular weight (kDa)
p-PI3K	abcam ab182651	1:500	85
p-AKT	CST 4060	1:1,000	60
PI3K	CST 13666	1:1,000	85
AKT	CST 4691	1:1,000	60
GLUT-2	Thermo Fisher 720238	1:1,000	57

### 5.11 Measurement of SCFA concentrations in cecum contents of mouse

The SCFA content of mouse cecum was determined with Agilent 7,890A gas chromatography (GC). The detection system was equipped with an Agilent J&W GC column (30 m × 320 μm × 0.25 μm) and a flame ionization detector (FID). The conditions were as follows: the initial column temperature was maintained at 100°C for 1 min and then increased to 150°C at a rate of 2°C/min for 5 min. The injector and detector temperatures were maintained at 270°C and 280°C, respectively. Hydrogen, air, and nitrogen were used as supplementary gases at flow rates of 30 ml/min, 4 ml/min, and 5 ml/min, respectively. The constant column flow + tail blow flow rate was 31.5 ml/min. The sample volume was 1 μl, and the column flow rate was 2 ml/min.

### 5.12 16S rDNA sequencing for gut microbiota

DNA was extracted from mouse cecum feces using the QIAamp DNA Stool Mini Kit (QIAGEN, Inc., Shanghai, China), and the purity and concentration of the extracted DNA were determined with 1% agarose gel electrophoresis. The primer sequences F: 5′-ACTCCTACGGGAGGCAGCAG-3′ and R: 5′-GGACTACHVGGGTWTCTAAI-3′ were used to amplify the V3-V4 hyper-variable region of the bacterial 16S rRNA gene (45). The samples were then sequenced in parallel utilizing Illumina MiSeq according to the user manual. The resulting raw data files were manipulated and filtered with the QIIME2 (version 1.8.0) software package. Raw sequences were imported into fastp QC (0.19.6^1^), and FLASH software (v1.2.11^2^) was used for pair-end double-end sequence splicing and screening for sequence optimization. Sequences with >97% similarity were clustered and annotated to generate operational taxonomic units (OTUs) using UPARSE software (version 7.0). Alpha diversity and microbial taxon distribution analyses were performed with QIIME2 software.

### 5.13 Statistical analysis

To compare the mean differences among the groups, one-way analysis of variance (ANOVA) by Duncan’s multiple range test was used. All data were expressed as mean ± SD, and *P* < 0.05 was considered statistically significant. All statistical analyses were performed using SPSS 20 software (SPSS Inc., Chicago, IL, USA).

## Data availability statement

The datasets presented in this study can be found in online repositories. The names of the repository/repositories and accession number(s) can be found in this article/Supplementary material.

## Ethics statement

The animal study was reviewed and approved by the Institutional Animal Care and Use Committee of the Zhejiang Academy of Agricultural Sciences.

## Author contributions

LC and GS conceived and designed the experiments. XX and CJ performed the experiments. HL, WH, JZ, SY, and CT analyzed the data. QJ wrote the manuscript. YF and ML helped perform the analysis and with constructive discussions. All authors read and approved the final manuscript.

## Footnotes

1
https://github.com/OpenGene/fastp

2
https://ccb.jhu.edu/software/FLASH/index.shtml
