# Supplementary material for: Antidiabetic effect of sciadonic acid on type 2 diabetic mice through activating the PI3K-AKT signaling pathway and altering intestinal flora
Source: Front Nutr. 2022 Dec 23;9:1053348. doi: 10.3389/fnut.2022.1053348 (PMC9816573; doi:10.3389/fnut.2022.1053348)
Supplement: Supplementary file 1 [file Table_1.DOCX]

**Antidiabetic effect of sciadonic acid on type 2 diabetic mice through activating the PI3K-AKT signaling pathway via altering intestinal flora**

Lin Chen^1^, Qihong Jiang^2^, Hongling Lu^1^,Chenkai Jiang^1^, Wenjun Hu^1^, Shaofang Yu^1^， Xingwei Xiang^2^, Chin Ping Tan^3,4^, Yongcai Feng^4^, Jianfang Zhang^4^, Mingqian Li^5*^, Guoxin Shen^1*^,

^1^Institute of Sericultural and Tea, Zhejiang Academy of Agricultural Sciences, Hangzhou, Zhejiang 310021, China;

^2^College of Food Science and Technology, Zhejiang University of Technology, Hangzhou, Zhejiang 310014, China;

^3^Department of Food Technology, Faculty of Food Science and Technology, University Putra Malaysia, 43400 Serdang, Malaysia.

^4^Xujing (Hangzhou) Biotechnology Research Institute Co., Ltd., Hangzhou, Zhejiang 310021, China;

^5^Cancer Institute of Integrated tradition Chinese and Western Medicine, Zhejiang Academy of Traditional Chinese Medicine, Tongde Hospital of Zhejiang Province, Hangzhou, Zhejiang, 310012, China;

*** Correspondence:**

Minqian Li

Address: Institute of Sericultural and Tea, Zhejiang Academy of Agricultural Sciences, Hangzhou Zhejiang 310021, China

Email: limingqian613@163.com

Cancer Institute of Integrated tradition Chinese and Western Medicine, Zhejiang Academy of Traditional Chinese Medicine, Tongde Hospital of Zhejiang Province, Hangzhou, Zhejiang, 310012, China;

Guoxin Shen

Address: Institute of Sericultural and Tea, Zhejiang Academy of Agricultural Sciences, Hangzhou Zhejiang 310021, China

Email: guoxin.shen@ttu.edu

**Supplemented data Figure S1.**


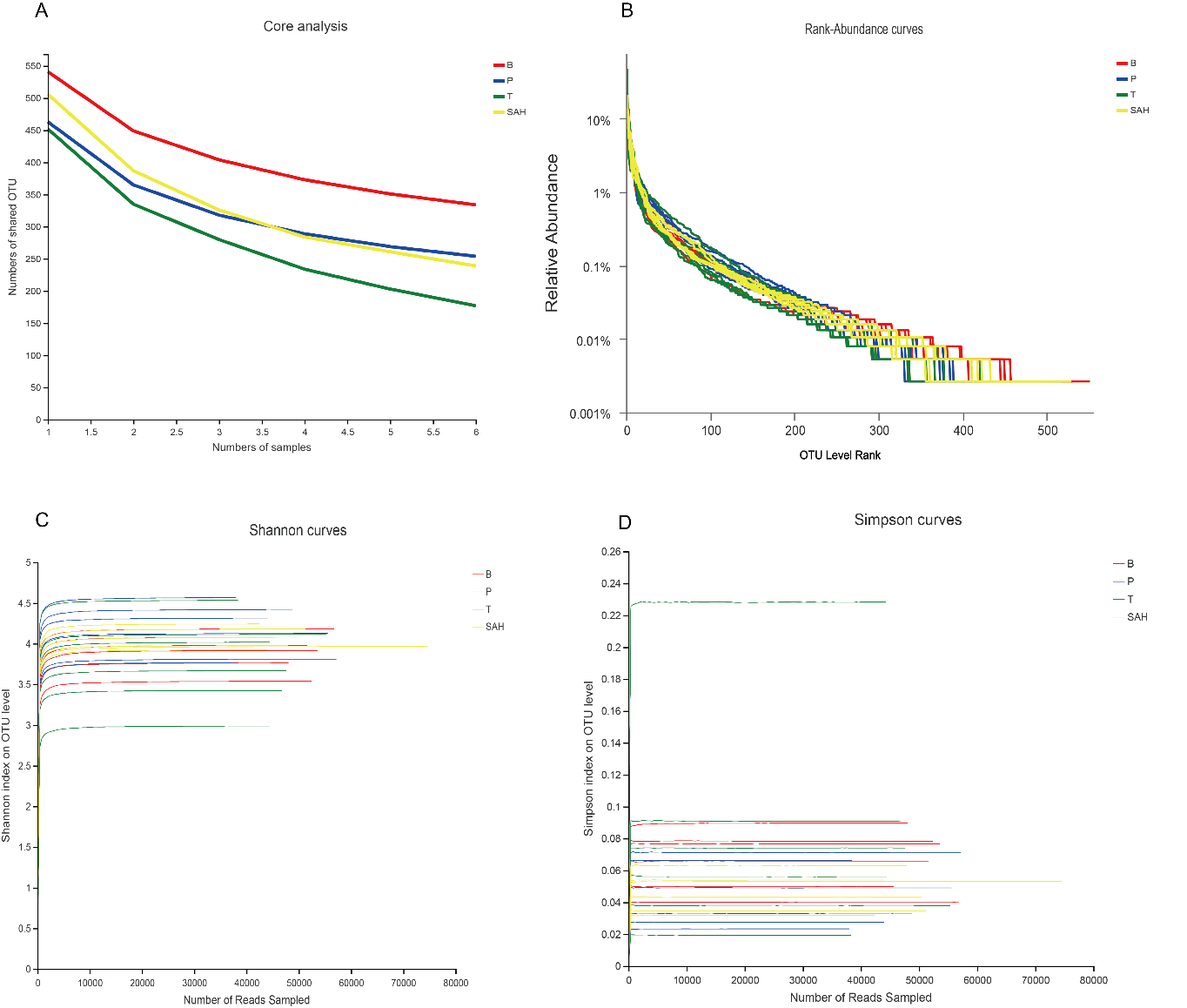


Figure s1. Core (A), rank-abundance (B), Shannon (C), and Simpson (D) rarefaction curves.
