# Supplementary material for: Antidiabetic effect of sciadonic acid on type 2 diabetic mice through activating the PI3K-AKT signaling pathway and altering intestinal flora
Source: Front Nutr. 2022 Dec 23;9:1053348. doi: 10.3389/fnut.2022.1053348 (PMC9816573; doi:10.3389/fnut.2022.1053348)
Supplement: Supplementary file 2 [file Table_2.DOCX]

Table S1 The ingredients of the normal and high fat diets.

|  | **HFD** | **NFD** |
| --- | --- | --- |
| **Ingredient** | **g** | **g** |
| Casein, 30 Mesh | 200 | 140 |
| L-Cystine | 3 | 1.8 |
| Corn Starch | 0 | 620 |
| Maltodextrin 10 | 125 | 35 |
| Sucrose | 68.8 | 100 |
| Cellulose, BW200 | 50 | 50 |
| Soybean Oil | 25 | 40 |
| Lard* | 245 | 0 |
| Mineral Mix S10026 | 10 | 35 |
| DiCalcium Phosphate | 13 | 13 |
| Calcium Carbonate | 5.5 | 5.5 |
| Potassium Citrate, 1 H2O | 16.5 | 16.5 |
| Vitamin Mix V10001 | 10 | 10 |
| Choline Bitartrate | 2 | 2.5 |
| **Total energy kcal/g** | **5.24** | **3.57** |
|  | kcal% | kcal% |
| Protein | 20 | 18.5 |
| Carbohydrate | 20 | 4.7 |
| Fat | 60 | 53.5 |
